# Vorinostat in combination with lenalidomide and dexamethasone in patients with relapsed or refractory multiple myeloma

**DOI:** 10.1038/bcj.2014.1

**Published:** 2014-02-21

**Authors:** D S Siegel, P Richardson, M Dimopoulos, P Moreau, C Mitsiades, D Weber, J Houp, C Gause, S Vuocolo, J Eid, T Graef, K C Anderson

**Affiliations:** 1 Myeloma Division, John Theurer Cancer Center, Hackensack University Medical Center, Hackensack, NJ, USA; 2 Dana-Farber Cancer Institute, Boston, MA, USA; 3 Department of Clinical Therapeutics, University of Athens, Athens, Greece; 4 University Hospital, Nantes, France; 5 MD Anderson Cancer Center, Houston, TX, USA; 6 Merck & Co. Inc., Whitehouse Station, NJ, USA; 7 Pharmacyclics Inc., Sunnyvale, CA, USA

**Keywords:** vorinostat, multiple myeloma, lenalidomide, refractory, relapsed

## Abstract

The addition of vorinostat to lenalidomide/dexamethasone represents a novel combination therapy in multiple myeloma (MM), informed by laboratory studies suggesting synergy. This was a phase I, multicenter, open-label, non-randomized, dose-escalating study in patients with relapsed or relapsed and refractory MM. Clinical evaluation, electrocardiogram, laboratory studies and adverse events were obtained and assessed. The maximum-tolerated dose was not reached owing to a non-occurrence of two dose-limiting toxicities per six patients tested at any of the dosing levels. Patients tolerated the highest dose tested (Level 5) and this was considered the maximum administered dose: at 400 mg vorinostat on days 1–7 and 15–21, 25 mg lenalidomide on days 1–21 and 40 mg dexamethasone on days 1, 8, 15 and 22, per 28-day cycle. Drug-related adverse events were reported in 90% of patients serious adverse experiences were reported in 45% of the patients and 22% of all patients had adverse experiences considered, possibly related to study drug by the investigators. A confirmed partial response or better was reported for 14/30 patients (47%) evaluable for efficacy, including 31% of patients previously treated with lenalidomide. Vorinostat in combination with lenalidomide and dexamethasone proved tolerable with appropriate supportive care, with encouraging activity observed.

## Introduction

Multiple myeloma (MM) is a relatively frequent hematologic malignancy characterized by the accumulation of clonal plasma cells. Despite recent improvements in treatment with significant increases in overall survival, the disease remains incurable in most patients.^1^ Following disease relapse, the mainstays of therapy for patients are immunomodulatory drugs (thalidomide, lenalidomide and pomalidomide), proteasome inhibitors (bortezomib and carfilzomib) and corticosteroids used singly, in combination with each other or in combination with other less active agents.^2, 3^ Moreover, the proteasome inhibitor carfilzomib and the immunomodulatory agent pomalidomide are now approved for the treatment of relapsed and refractory MM, providing new options for this particularly vulnerable group of patients.^4, 5, 6^ Nonetheless, despite this continuum of advances, relapsed and refractory MM patients remain an unmet medical need, and combination approaches provide a mainstay of management.^2^ Data suggest that the addition of histone deacetylase (HDAC) inhibitors to treatment regimens can synergistically increase the activity of anticancer agents in both MM and other tumor types.^7, 8^ HDAC inhibition leads to hyperacetylation of nucleosomal histones and a corresponding inhibition in the transcription of certain genes, some of which are involved in cell proliferation. This can result in a phenotypic alteration of transformed cells, including growth arrest, apoptosis or senescence.^9^ Vorinostat is a potent broad oral HDAC inhibitor that binds directly to the catalytic pocket of HDAC enzymes^10, 11^ and was approved in the United States in 2006 for the treatment of patients with cutaneous T-cell lymphoma.^12^ Previous studies have shown that vorinostat is a potent inducer of apoptosis in MM cells.^13^ Along with several other oncologic indications, vorinostat has been tested in MM both as monotherapy, where it has achieved stable disease, and in combination regimens.^13, 14, 15^ When combined with bortezomib, it can achieve enhanced inhibition of protein breakdown^16^ and increased responses in clinical trials.^17^ Another oral HDAC inhibitor (panobinostat) combined with bortezomib achieves similar responses in relapsed refractory MM.^18^ In this report, we present the results from a phase I dose escalation study to assess the safety and tolerability of a combination regimen of vorinostat, combined with lenalidomide and dexamethasone, in patients with relapsed or refractory MM.

## Materials and Methods

### Study design

This was a phase I, multicenter, open-label, non-randomized, dose-escalating study in patients with relapsed or relapsed and refractory MM (NCT no. NCT00642954). Patients who met all the inclusion/exclusion criteria and who signed the informed consent form were assigned an allocation number. Patients were treated with vorinostat in combination with lenalidomide and dexamethasone in 28-day treatment cycles. Three dose levels were used in this study to determine the maximum-tolerated dose (MTD) for the combination regimen. At least three patients were entered at each dose level. Dose-limiting toxicities (DLTs) were counted during the first treatment cycle only. New dose levels began accrual only after all patients at the current dose level were observed for at least one cycle. The Principal Investigator at each site consulted with the sponsor to determine the appropriate dose level for a new patient. Patients received up to eight cycles or until unacceptable toxicity, disease progression or the withdrawal of consent. Patients who did not have disease progression and continued to meet the eligibility criteria with satisfactory tolerance after eight cycles were offered continued treatment at the same dose level in an extension study.

### Eligibility criteria

Patients ⩾18 years of age on the day of signing informed consent with an established diagnosis of MM based on the standard myeloma diagnostic criteria were eligible for enrollment. Patients must have had measurable relapsed or relapsed and refractory MM after the most recent treatment regimen as per the European Group for Blood and Marrow Transplantation criteria, and must have also had performance status of ⩽2 on the Eastern Cooperative Oncology Group Performance Scale. All patients agreed to follow the regional requirements for lenalidomide counseling, pregnancy testing and birth control, and be willing and able to comply with the regional requirements (e.g., periodic pregnancy tests and safety labs).

### Treatments

Patients were assigned to the dose cohort open at the time of individual enrollment. The doses of study medications were escalated as shown in Table 1. At each dose level, three evaluable patients were enrolled, treated and observed for one full cycle. Dose escalation proceeded in the absence of observed DLTs. Treatment was administered on an outpatient basis: one treatment cycle was 28 days long. Cycles 1–8 were considered the base protocol, whereas cycles beyond that were considered extension. Vorinostat and dexamethasone were dispensed on day 1 of each 28-day cycle when the patient arrived for the clinical visit. Lenalidomide was prescribed through and in compliance with regional requirements for both US sites and European sites.

Vorinostat was taken within 30 min of a meal, if possible. Patients were advised to take all three study medications at approximately the same time each day on an ongoing basis. For all three study drugs, there were no substitutions for doses that were missed, coughed up or vomited. The use of low dose (e.g., 81 mg q.d.) acetyl salicylic acid was recommended to provide prophylaxis against thromboembolic events during protocol therapy.

In the absence of treatment delays owing to adverse events (AEs) and if the patient had experienced a response or clinical benefit, treatment with vorinostat in combination with lenalidomide and dexamethasone may have continued until one of several criteria was applied: progressive disease, intercurrent illness that prevented further administration of treatment, unacceptable adverse experiences, patient withdrew consent, if in the opinion of the investigator, a change of therapy would be in the best interest of the patient, general or specific changes in the patient's condition that rendered the patients ineligible for further treatment, non-compliance with study medication or protocol-required evaluations and study visits, and patient was lost to follow-up.

### Measurements

The MTD was defined as the highest tested dose at which six patients had been treated with no more than one out of six patients experiencing a DLT within the first cycle of therapy. Hematologic DLTs were defined as either grade 4 neutropenia lasting for ⩾7 days in duration, any grade 4 thrombocytopenia or any grade 5 hematologic toxicity. Non-hematologic DLT was defined as any grade 3 or higher non-hematologic toxicity with specific exceptions detailed in the protocol.

Overall safety measurements, including physical examination, vital signs, electrocardiogram, determination of Eastern Cooperative Oncology Group performance status, laboratory studies and adverse experiences, were obtained and assessed before drug administration, and at designated intervals throughout the study.

Response to study therapy was assessed using the modified European Society for Blood and Marrow Transplantation criteria including complete response, near complete response, very good partial response (VGPR), partial response (PR), minimal response (MR), stable disease (SD)/no change and progressive disease. This was used to estimate response rate (RR), time to response, response duration and time to progression for the combination regimen.

### Statistical methodology

The primary objective of this study was to determine the MTD for the combination regimen of vorinostat, lenalidomide and dexamethasone in patients with relapsed or refractory MM. This objective was addressed by evaluating DLT data and other safety data.

The 3+3 design was used. The number of patients enrolled into each dose level, 3–6, was the standard number used to determine the MTD. Once the MTD was established, additional patients up to a total of 14 patients were to be enrolled at the MTD to confirm safety and tolerability. The maximum number of patients was 44. With 14 patients, the upper bound of the 80% confidence interval for DLT rate excluded a rate of 33% if two or fewer patients developed DLTs.

The safety analyses were based on all patients as treated (APaT) population. This population consisted of all enrolled patients who had received at least one dose of study medication. The efficacy analyses were based on evaluable patients with appropriate available measurements for response. All analyses were generated as summaries or listings. No other statistical hypothesis tests were performed. For the binary end points, the exact method was used when calculating the confidence intervals. For the time-to-event end points, the Kaplan–Meier method was used when calculating the median.

## Results

**Figure 1:**
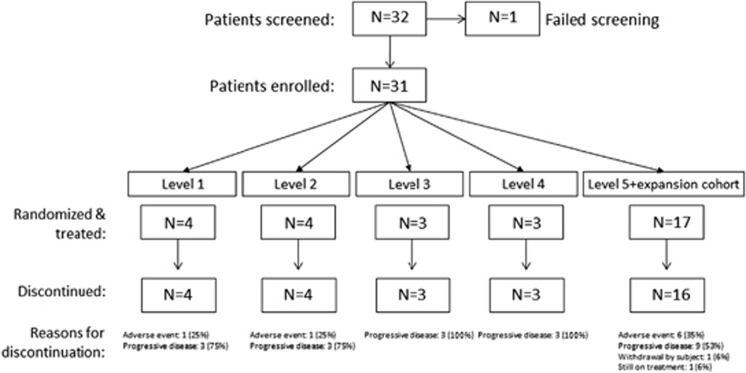
CONSORT diagram.

There were 32 patients screened for inclusion into this study, with one patient excluded during screening as a result of failing to meet the inclusion criteria for renal function (Figure 1). A total of 31 patients were therefore enrolled at five sites in France, Greece and the United States. Patient characteristics for all enrolled patients are listed in Table 2.

A summary of best confirmed responses is presented in Table 3. One patient was not evaluable for efficacy because study treatment was discontinued without any postbaseline assessments of response. The overall RR (PR or better) for the study (best confirmed response) was 47%. The overall RR was 43% for the 14 patients in the dose escalation cohort (DEC) and 50% for the 16 patients in the maximum planned dose (MPD) cohort. The median time to response for the DEC was 91 days (range: 29–499 days) and for the MPD it was 57 days (range: 29–86 days). The median duration of response for the DEC was 134 days (range: 106–302 days) and for the MPD it was 139 days (range: 97–547 days). The clinical benefit rate (MR or better) for all patients (best confirmed response) was 57%, and 63% and 50% for the MPD and DEC, respectively.

**Figure 2:**
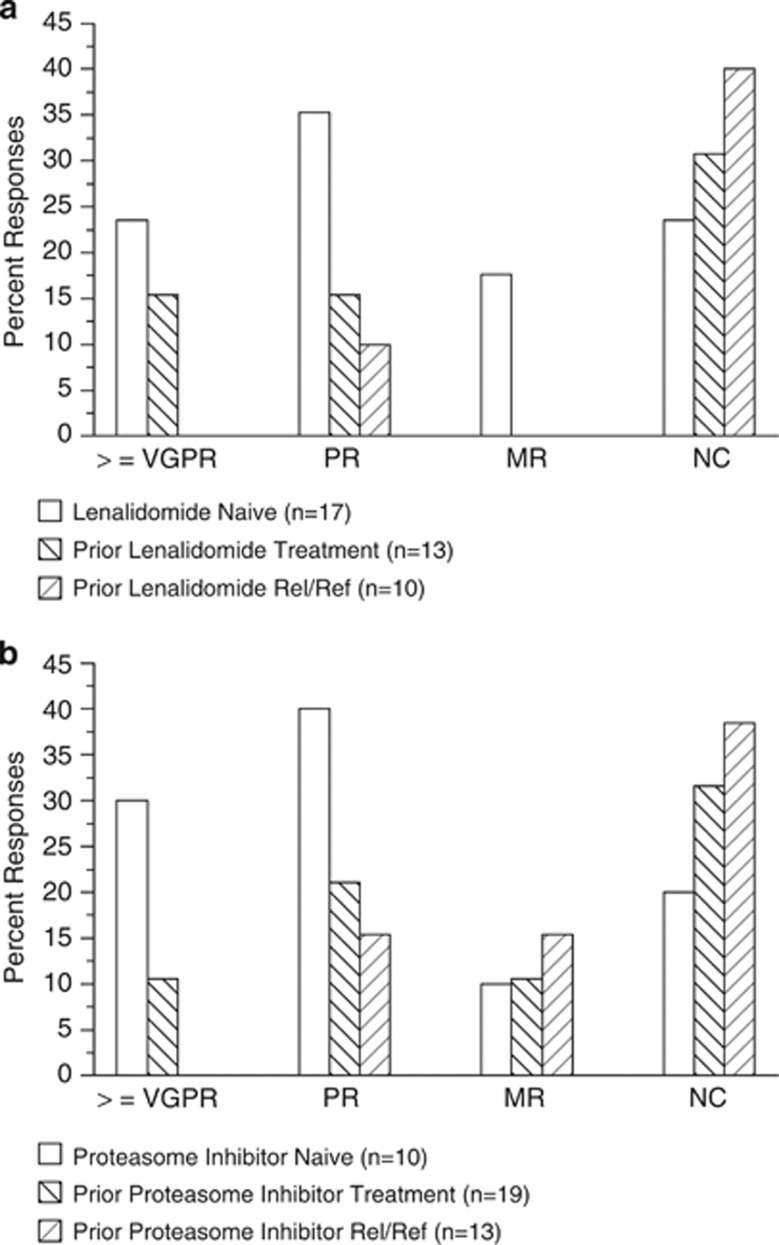
(**a**) Confirmed responses by prior lenalidomide history. (**b**) Confirmed responses by prior proteasome inhibitor history.

Confirmed responses with respect to lenalidomide status are presented in Figure 2a. Among patients who had not received lenalidomide treatment before enrolling in this study (lenalidomide naive), 24% had a confirmed response of VGPR or better, 35% had a confirmed response of PR, 18% had a confirmed response of MR and 24% had SD. For patients who had received prior lenalidomide treatment for myeloma, 15% had a confirmed response of VGPR or better, and an additional 15% had a confirmed response of PR, with 31% having SD; in patients whose disease was considered relapsed and refractory to prior lenalidomide treatment, 10% had a confirmed response of PR and 40% had SD.

Confirmed responses with respect to prior proteasome inhibitor treatment are presented in Figure 2b. In patients who had not received proteasome inhibitor treatment before enrolling in this study (proteasome inhibitor naive; *n*=10), 30% had a confirmed response of VGPR or better, 40% had a confirmed response of PR, 10% had a confirmed response of MR and 20% had SD. For patients who had received prior proteasome inhibitor treatment for myeloma (*n*=19), 11% had a confirmed response of VGPR or better, 21% had a confirmed response of PR, 11% had a confirmed response of MR and 32% had SD. Among patients whose disease was considered relapsed and refractory to prior proteasome inhibitor treatment (*n*=13), 15% had a confirmed response of PR, 15% had a confirmed response of MR and 39% had SD.

All 31 patients (100%) enrolled in this study experienced at least one AE (Table 4). In total, 28 (90%) patients had at least one AE that was considered possibly treatment-related. A total of 14 patients (45%) experienced at least one serious AE during study treatment (six in the DEC and eight in the MPD cohort), of which 7 were considered related to therapy. A higher percentage of patients in the MPD cohort experienced serious drug-related events (5 patients (29%) versus 2 patients (14%)), discontinued study treatment owing to an AE (7 patients (41%) versus 3 patients (21%)) or drug-related AE (4 patients (24%) versus 2 patients (14%)) as compared with the DEC group. The most commonly reported AEs included anemia (58% 64% in the DEC and 53% in the MPD cohort), thrombocytopenia (58% 43% in the DEC and 71% in the MPD cohort), diarrhea (55% 64% in the DEC and 47% in the MPD cohort), fatigue (55% 43% in the DEC and 65% in the MPD cohort) and cough (45% 50% in the DEC and 41% in the MPD cohort), but generally proved manageable with dose reduction and supportive care.

## Discussion

This was an open-label, multicenter, non-randomized phase I dose escalation study designed to determine the MTD for the all-oral combination regimen of vorinostat, lenalidomide and dexamethasone in patients with relapsed or relapsed and refractory MM. Secondary objectives included safety and tolerability, with the exploratory objective of evaluation of clinical activity for this novel combination regimen. This report demonstrates that the MTD for the combination regimen of vorinostat, lenalidomide and dexamethasone was not reached owing to non-occurrence of two DLTs per six patients at any of the tested dose levels. Patients tolerated the highest administered dose level in the study (400 mg vorinostat on days 1–7 and days 15–21, 25 mg lenalidomide on days 1–21 and 40 mg dexamethasone on days 1, 8, 15 and 22), which was considered the recommended phase II dose. A higher proportion of serious AEs and discontinuations owing to AE were seen in the highest administered dose level as compared with the DECs. However, vorinostat in combination with lenalidomide and dexamethasone can thus be considered generally tolerable with appropriate supportive care.

Given the progressively lower RRs to treatment following relapse in patients with MM, the fact that 31% of patients previously treated with lenalidomide and 32% of patients previously treated with proteasome inhibitors had confirmed responses of PR or better supports the hypothesis that the combination regimen of vorinostat, lenalidomide and dexamethasone is active in patients with relapsed or relapsed and refractory MM. Although further research is required to delineate the precise mechanism of action underlying the clinical activity observed with the combination of vorinostat, lenalidomide and dexamethasone, interesting results have been seen with other HDAC combinations in the treatment of MM. Triple combinations of the HDAC panobinostat with dexamethasone and either bortezomib or lenalidomide have demonstrated activity greater than combinations of HDAC and dexamethasone alone.^18^ Deregulation of genes resulting from these triple combinations was also shown to be different and separate from deregulation seen with monotherapy.^13^ More selective HDAC 6 inhibitors have also shown synergistic activity with proteasome inhibitors and lenalidomide, with the potential for improved tolerability.^19^

Several promising and rationally designed therapies in the treatment of MM include the combination of the proteasome inhibitors bortezomib or carfilzomib with lenalidomide and dexamethasone.^20^ Dose escalation trials in pretreated populations of MM patients proved promising, but with higher rates of severe AEs reported in the carfilzomib study. In addition, a retrospective chart review of 25 lenalidomide/dexamethasone refractory patients treated with the combination of lenalidomide, vorinostat and dexamethasone showed a VGPR rate of 4%, a PR rate of 24% and an MR rate of 20% for an ORR of 28% and CBR of 48%.^21^ Further data on combination therapy in MM should be forthcoming.

Overall, the combination of vorinostat with lenalidomide and dexamethasone had an acceptable safety profile at the MTD. In addition, this combination regimen resulted in clinical responses in a heavily pretreated population of relapsed or relapsed and refractory MM. Although responses were generally better in lenalidomide or proteasome inhibitor-naive populations, clinical activity was observed in lenalidomide- and proteasome inhibitor-treated patients. Patients treated at the MTD had a higher rate of toxicity (although still generally tolerable) than did patients treated in the DEC, but showed a trend of higher RRs, shorter times to response and a longer duration of response, suggesting that this regimen justifies further evaluation in relapsed or refractory MM. Further research will define the role of this combination in this setting and among less heavily pretreated patients.

## Figures and Tables

**Table 1:** Dosing regimen

*Dose level no.*	*Vorinostat dose (mg q.d.) 7 days on 7 days off (days 1–7 and days 15–21) in each 28-day cycle*	*Lenalidomide dose (mg q.d.) × 21 days (days 1–21) in each 28-day cycle*	*Dexamethasone dose (mg q.d.) on days 1, 8, 15 and 22 in each 28-day cycle*
1	300	10	40
2	400	10	40
3	400	15	40
4	400	20	40
5	400	25	40

**Table 2:** Patient characteristics

	*Levels 1–4*	*Level 5+ expansion cohort*	*Total*
	n *(%)*	n *(%)*	n *(%)*
Patients in population	14	17	31
			
*Gender*			
Male	8 (57.1)	10 (58.8)	18 (58.1)
Female	6 (42.9)	7 (41.2)	13 (41.9)
			
*Age (years)*			
Patients with data	14	17	31
Mean	62.7	64.1	63.5
S.d.	6.8	7.2	7.0
Median	61.0	64.0	63.0
Range	53–74	52–79	52–79
			
*Race*			
White	14 (100.0)	16 (94.1)	30 (96.8)
Black	0 (0.0)	1 (5.9)	1 (3.2)
			
*Myeloma type—heavy chain class*			
IgG	6 (42.9)	9 (52.9)	15 (48.4)
IgA	6 (42.9)	4 (23.5)	10 (32.3)
Not detected	2 (14.3)	4 (23.5)	6 (19.4)
			
*Myeloma type—light chain class*			
Lambda	6 (42.9)	3 (17.6)	9 (29.0)
Kappa	8 (57.1)	14 (82.4)	22 (71.0)
			
*ISS stage at screening,* n *(%)*			
I	9 (64.3)	7 (41.2)	16 (51.6)
II	5 (35.7)	9 (52.9)	14 (45.2)
III	0 (0.0)	1 (5.9)	1 (3.2)
			
*Baseline ECOG,* n *(%)*			
0	6 (42.9)	5 (29.4)	11 (35.5)
1	7 (50.0)	10 (58.8)	17 (54.8)
2	1 (7.1)	2 (11.8)	3 (9.7)
			
*Number of prior treatment regimens*			
Patients with data	14	17	31
			
*Number of prior treatment regimens*			
Mean	3.1	4.4	3.8
S.d.	1.4	2.5	2.2
Median	2.5	4.0	4.0
Range	1–6	2–10	1–10
			
*Prior exposure to lenalidomide,* n *(%)*			
Yes	8 (57.1)	6 (35.3)	14 (45.2)
No	6 (42.9)	11 (64.7)	17 (54.8)
			
*Prior exposure to thalidomide,* n *(%)*			
Yes	8 (57.1)	13 (76.5)	21 (67.7)
No	6 (42.9)	4 (23.5)	10 (32.3)
			
*Prior exposure to proteosome inhibitor,* n *(%)*			
Yes	7 (50.0)	13 (76.5)	20 (64.5)
No	7 (50.0)	4 (23.5)	11 (35.5)
			
*Transplant history*			
Patients with ⩾1 prior transplant	11 (78.6)	14 (82.4)	25 (80.6)
Patients with no prior transplant	3 (21.4)	3 (17.6)	6 (19.4)

Abbreviations: ECOG, Eastern Cooperative Oncology Group; Ig, immunoglobulin; ISS, International Staging System.

Subjects with missing baseline information are excluded from the corresponding analysis ISS staging derived from screening values of β2-microglobulin and albumin.

**Table 3:** Best confirmed response summary (patients with efficacy evaluation)

	*Levels 1–4*		*Level 5+ expansion cohort*		*Total*	
	n	*% (95% CI)*	n	*% (95% CI)*	n	*% (95% CI)*
Number of patients in population	14		16		30	
						
*Number (%) of patients with best response*						
Complete response	0	0.0 (0.0, 23.1)	1	6.3 (0.15, 30.2)	1	3.3 (0.08, 17.2)
Near complete response	1	7.1 (0.18, 33.8)	0	0.0 (0.0, 20.5)	1	3.3 (0.08, 17.2)
Very good PR	2	14.3 (1.77, 42.8)	2	12.5 (1.55, 38.3)	4	13.3 (3.75, 30.7)
PR	3	21.4 (4.6, 50.7)	5	31.3 (11.0, 58.6)	8	26.7 (12.2, 45.8)
Minimal response	1	7.1 (0.18, 33.8)	2	12.5 (1.55, 38.3)	3	10.0 (2.1, 26.5)
Stable disease	3	21.4 (4.6, 50.7)	5	31.3 (11.0, 58.6)	8	26.7 (12.2, 45.8)
Progressive disease	4	28.6 (8.38, 58.1)	1	6.3 (0.15, 30.2)	5	16.7 (5.64, 34.7)
						
	*Days (95% CI)*		*Days (95% CI)*		*Days (95% CI)*	
Median time to response (PR or better)	91 (29, 499)		57 (29, 86)		57 (29, 113)	
Median duration of response (PR or better)	134 (106, 302)		139 (97, 547)		139 (106, 302)	
25% Time to response (PR or better)	29 (22, 113)		33 (29, 57)		29 (29, 57)	
25% Duration of response (PR or better)	106 (72, 148)		102 (85, 143)		106 (85, 143)	
75% Time to response (PR or better)	499 (68, 617)		72 (57, 358)		113 (57, 499)	
75% Duration of response (PR or better)	302 (120, 386)		401 (134, 814)		302 (134, 547)	

Abbreviations: CI, confidence interval; PR, partial response.

Excludes one patient who discontinued without any postbaseline efficacy assessments.

**Table 4:** Adverse event summary

	*Levels 1–4*	*Level 5+ expansion cohort*	*Total*
	n *(%)*	n *(%)*	n *(%)*
Patients in population	14	17	31
With one or more adverse events	14 (100.0)	17 (100.0)	31 (100.0)
With no adverse event	0 (0.0)	0 (0.0)	0 (0.0)
With drug-related^a^ adverse events	11 (78.6)	17 (100.0)	28 (90.3)
With serious adverse events	6 (42.9)	8 (47.1)	14 (45.2)
With serious drug-related adverse events	2 (14.3)	5 (29.4)	7 (22.6)
Who died	1 (7.1)	3 (17.6)	4 (12.9)
Discontinued^b^ owing to an adverse event	3 (21.4)	7 (41.2)	10 (32.3)
Discontinued owing to a drug-related adverse event	2 (14.3)	4 (23.5)	6 (19.4)
Discontinued owing to a serious adverse event	1 (7.1)	3 (17.6)	4 (12.9)
Discontinued owing to a serious drug-related adverse event	0 (0.0)	0 (0.0)	0 (0.0)

a Determined by the investigator to be related to the drug.

b Study medication withdrawn.

## Notes

DSS reports working as a consultant for Merck, Millennium and Onyx and having served on speakers' bureaus for Celgene, Millennium and Onyx. PR reports serving on advisory committees at Celgene, Johnson & Johnson and Millennium. MD has received consulting fees from Celgene, Orthobiotech and Onyx. PM reports board memberships at Celgene, Janssen and Millennium. CM reports receiving consultancy fees from Merck & Co., Millennium, Celgene, Novartis, Johnson & Johnson, Arno Therapeutics, Bristol-Myers Squibb and Curis. He is also involved in patents and has received royalties from PharmaMar. JH, CG, SV, JEE and TG are Merck employees and may own stock in the company. KCA reports board memberships at Acetylon and Oncopep and consulting for Celgene, Onyx, Gilead and Sanofi-Aventis.
